# Differential Demographic and Clinical Characteristics between MMR Vaccinated and Unvaccinated Children in South Korea: A Nationwide Study

**DOI:** 10.3390/vaccines9060653

**Published:** 2021-06-15

**Authors:** Dongwon Yoon, Juhwan Kim, Juyoung Shin

**Affiliations:** 1 School of Pharmacy, Sungkyunkwan University, Suwon 16419, Korea; dwyoon09@gmail.com (D.Y.); napa928@hotmail.com (J.K.); 2 Samsung Advanced Institute for Health Sciences & Technology, Sungkyunkwan University, Seoul 06355, Korea

**Keywords:** measles, measles-mumps-rubella vaccine, vaccine uptake, vaccine hesitancy

## Abstract

In the context of recent measles outbreaks, substantial factors associated with measles-mumps-rubella (MMR) unvaccination need to be clarified. This study aimed to identify differential demographic and clinical characteristics between MMR vaccinated and unvaccinated groups. We used a large-linked database to identify children born between 2008 and 2016 by combining data from the Korea Immunization Registry Information System and National Health Information database. The MMR vaccination status was ascertained up to the age of 2 to define MMR vaccinated and unvaccinated groups. We conducted a multivariate logistic regression to estimate odds ratios (ORs) with 95% confidence intervals (CIs) to identify factors associated with MMR unvaccination. Of 3,973,253 children, 75,674 (1.9%) did not receive the MMR vaccine. Compared with the MMR vaccinated group, the underutilization of healthcare resources was more notable in the MMR unvaccinated group (number of outpatient visits (5.73 ± 12.1 vs. 25.8 ± 17.06); days hospitalized (1.69 ± 14.5 vs. 2.32 ± 6.90)). Children were less likely to receive the MMR vaccine if they were born with congenital anomaly (OR 2.12; 95% CI 1.90–2.36), were never admitted to an intensive care unit (1.88; 1.78–1.98), or never visited an emergency room (3.57; 3.53–3.72). There were substantial factors associated with MMR unvaccination, underscoring a need to optimize targeted interventions tailored to the subset of children in South Korea.

## 1. Introduction

Historically, measles accounted for the leading cause of death in children with its elimination being one of the top global priorities [1]. Following the introduction of the measles-containing vaccine, a tremendous effort made by the health authorities as well as healthcare practitioners in promoting its uptake led to the declaration of measles elimination in several countries. However, despite such an effort, a recent resurgence of measles outbreaks has taken its toll on the global health burden [2]. In South Korea, the most recent measles outbreak occurred between 2018 and 2019. There were 48 out of 194 cases involving children aged less than nine years old, of which 38 did not receive and 10 only received one dose of the measles-mumps-rubella (MMR) vaccine [3]. Given that all identified cases were either partially or not vaccinated, maintaining a high vaccination coverage is strongly recommended to prevent a measles outbreak.

While MMR vaccination coverage in South Korea is estimated to be over 95%, intermittent community outbreaks of measles imply a subpopulation still not inoculated with the MMR vaccine [4,5,6]; these unvaccinated individuals are likely to have differential characteristics and to be a threat to public health and a major barrier to herd immunity. Several past studies have shown that factors such as living in a household with other children, the birth order, being a single parent, having a young maternal age, having a high parental education level and unemployment were associated with MMR unvaccination [7,8,9,10,11]. However, these studies were either based on a survey involving a small number of participants from a part of a region or focused only on the parental demographic and socioeconomic factors.

In this context, there is a need to explore these factors in the wider population, focusing not only on the parental but also child-specific characteristics. Therefore, we aimed to investigate the differential demographic and clinical characteristics between MMR vaccinated and unvaccinated children using a nationwide database in South Korea. For the exploratory objective, we assessed the uptakes of other routinely recommended vaccines among those who did not receive the MMR vaccine.

## 2. Materials and Methods

### 2.1. Data Source

We constructed a large-linked database to identify children born between January 2008 and December 2016 by combining data from the Korea Immunization Registry Information System (KIRIS) and the National Health Information Database (NHID) using an anonymized identifier. Data on the type of vaccine, date of immunization, age at immunization, dosing schedule information and immunization site were retrieved from the KIRIS, and data on diagnosis information, prescription, treatment or procedure information, medical insurance type, income level and location of residence were retrieved from the NHID.

The NHID is a nationwide database including the entire 50 million population of South Korea. The database includes individual demographic information as well as healthcare utilization information regarding records on in- and outpatient healthcare usage (disease diagnosis, length of hospital admission, procedures received, costs of treatment) and prescription records (drugs received, days of prescription, dosage) under the fee-for-service reimbursement system in South Korea. Diagnosis information are coded by the International Classification of Diseases, 10th Revision (ICD-10) and have been validated in past studies that reported a positive predictive value of 82% [12,13]. Both prescription and procedure information were coded by the national codes. By using this nationwide linked database, we can secure demographic and clinical information from children in Korea, and the effect of recall bias can be minimized.

### 2.2. Study Population

Our study population included children aged 0 to 2 years born between 2008 and 2016. The study period was determined to take into account the implementation of complimentary MMR vaccination and administrative delays in processing the accumulated data. Considering the possibility of not being immunized with the MMR vaccine due to death, we excluded children who died before reaching the age of 2. In addition, to prevent the potential misclassification of the MMR vaccination status, we adopted a rigorous criterion by excluding those who received their first dose of MMR vaccine beyond the age of 2 (out-of-schedule) or those who only had a record of the second dose of MMR vaccine (missing information on the first dose of MMR vaccine).

### 2.3. Ascertainment of MMR Vaccination Status 

In South Korea, detailed information on immunization is reported by the healthcare practitioners in order to request the reimbursement of immunization fees from the health authority for all vaccines included in the National Immunization Program (NIP). MMR vaccination has been covered free-of-charge since 2009, thereby enabling a precise exposure ascertainment during the study period. While NIP recommends MMR vaccination at 12–15 months, the MMR vaccination status was assessed up to the first two years of birth to account for potential delayed immunization in the KIRIS. The MMR vaccinated group included those who had received at least one dose of MMR vaccine by the age of 2, and the MMR unvaccinated group included those without any MMR vaccination record in the database. 

The index date was defined as the date of MMR vaccination for the MMR vaccinated group. For the MMR unvaccinated group, the index date was defined as the midyear (i.e., July 1st) of the subsequent birth year (i.e., the index date for an MMR unvaccinated child born in 2010 was 1 July 2011) of each individual. This definition can secure a comparability between the MMR vaccinated and MMR unvaccinated groups because the average age at the index date would be similar in those two groups considering the recommended dosing schedule of the MMR vaccine.

### 2.4. Factors Associated with MMR Unvaccination

We assessed both parental and children characteristics from the database. Parental factors such as residence type, type of health insurance and income level were assessed at the index date. The type of health insurance was divided into three groups: employer-sponsored, self-employed and medical aid beneficiary. For child-specific factors (i.e., factors of the vaccinee), we assessed the comorbidities and healthcare utilization of each individual. To examine whether the health status of children was associated with MMR unvaccination, we included birth injury, congenital anomaly, infections in perinatal period, low birth weight, small for gestational age, asplenia, history of anaphylaxis, prior MMR infection and cancer. Each comorbidity was examined by the ICD-10 codes listed in Supplementary Table S1. Healthcare utilization was measured by counting the numbers of outpatient visits, days of hospitalization, intensive care unit (ICU) visits, emergency room (ER) visits and the Korean traditional medicine clinic visits. The comorbidities and healthcare utilization were assessed during a 12-month period prior to the index date.

### 2.5. Statistical Analyses

We calculated the mean and standard deviation (SD) for continuous variables and presented frequencies and proportions for categorical variables. To identify factors associated with MMR unvaccination, we conducted a multivariate logistic regression to estimate odds ratios (ORs) with 95% confidence intervals (CIs). We adjusted for sex, residence type, type of health insurance, income level and predefined comorbidities in the regression model. For the exploratory objective, we assessed the immunization records of other routinely administered vaccines (e.g., Bacillus Calmette–Guérin, Hepatitis B, Diphtheria-Tetanus-acellular pertussis, inactivated poliovirus, Haemophilus influenzae, pneumococcal vaccine) recommended prior to the date of MMR vaccination, stratified by the MMR vaccination status. All analyses were performed using SAS Enterprise Guide 7.1 for Windows (SAS Institute Inc., Cary, NC, USA). A two-tailed *p*-value < 0.05 was considered statistically significant.

### 2.6. Ethical Approval and Patient Involvement

This study was reviewed and approved by the Institutional Review Board of Sungkyunkwan University (Approval number: SKKU 2021-03-011), and informed consent was waived as only de-identified data were used in our analyses.

## 3. Results

### 3.1. Population in Study Cohort

**Figure 1:**
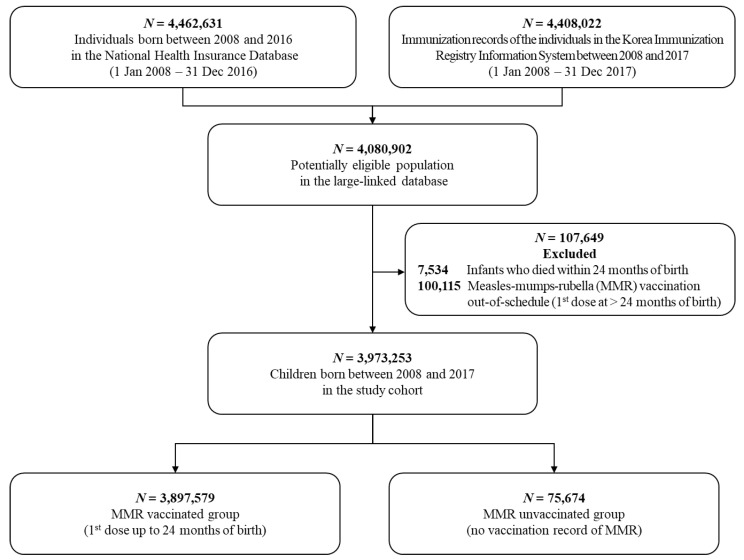
Flow chart of the study cohort construction in the large-linked database of the Korea Immunization Registry Information System (KIRIS) and National Health Information Database (NHID) between January 2008 and December 2016.

The study population included 3,973,253 children born between 2008 and 2016 with at least 24 months of healthcare coverage. Of them, 3,897,579 (98.1%) received at least one dose and 75,674 (1.9%) did not receive any dose of the MMR vaccine during the first two years of birth (Figure 1). The baseline characteristics by MMR vaccination status are presented in Table 1. There were more boys in the MMR vaccinated group (51.3%) and girls in the MMR unvaccinated group (50.3%). For the parental socioeconomic status, we observed considerable differences in the type of health insurance and household income level. In the MMR vaccinated group, employer-sponsored health insurance accounted for the majority of the group (68.1%), and self-employed health insurance only accounted for 20.1%. However, in the MMR unvaccinated group, the proportion of self-employed health insurance was 57.8%, and employer-sponsored health insurance only accounted for 27.9%. There was a higher proportion with a low household income level in the MMR unvaccinated group (21.1%) versus the vaccinated group (7.3%), and the majority of the vaccinated group had upper-middle or high household income levels.

Several comorbidities also showed significant differences. Compared with the MMR vaccinated group, congenital anomaly and cancer were more prevalent in the MMR unvaccinated group. However, birth injury, infections in perinatal period, low birth weight, small for gestational age, asplenia, history of anaphylaxis and prior MMR infection were relatively higher in the MMR vaccinated group. Underutilization of healthcare resources was more notable in the MMR unvaccinated group compared with the MMR vaccinated group (number of outpatient visits (5.73 ± 12.1 vs. 25.8 ± 17.06); days hospitalized (1.69 ± 14.5 vs. 2.32 ± 6.90)). In addition, children who had ICU or ER visits were more prevalent in the MMR vaccinated group (4.56% and 26.9%, respectively) compared with the MMR unvaccinated group (1.94% and 8.63%, respectively).

### 3.2. Annual Trends of Vaccine Coverage

**Figure 2:**
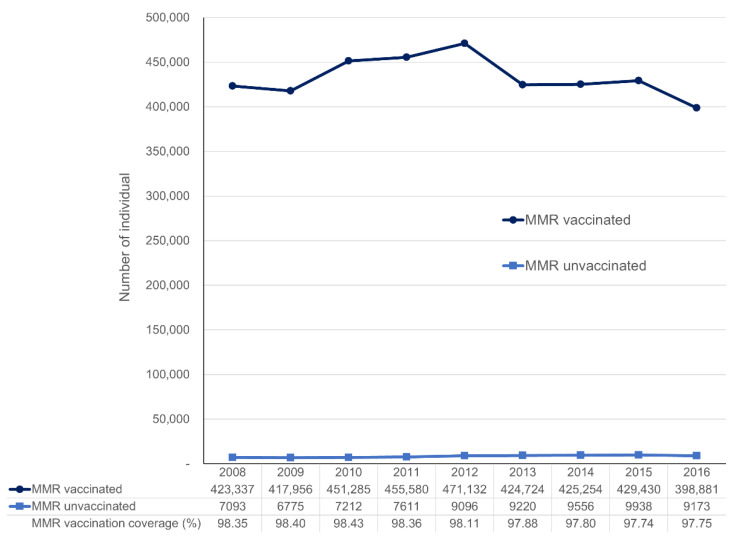
Annual MMR vaccine coverage and the numbers of vaccinated and unvaccinated children born between January 2008 and December 2016. Abbreviations: MMR, Measles-Mumps-Rubella.

The average MMR vaccine coverage was 98.1% in our study population; the annual rate ranged between 97.7% and 98.4%, declining slightly as the number of MMR unvaccinated children increased throughout the study period (Figure 2). In addition, the exploratory analysis showed that the overall coverage of other vaccines in the MMR unvaccinated group remained below 50% throughout the study period despite the increasing trend with time (Supplementary Figure S1).

### 3.3. Factors Associated with MMR Unvaccination

The demographic and clinical factors associated with MMR unvaccination are present in Table 2. Girls were less likely to receive the MMR vaccine on schedule with an adjusted odds ratio (aOR) of 1.02 (95% CI, 1.00 to 1.03). Parental socioeconomic factors including a low household income level (aOR 3.66; 95% CI, 3.58 to 3.74), self-employed health insurance (7.34; 7.21 to 7.46) or residing in a metropolitan area (1.44; 1.41 to 1.46) were more likely to be observed in the MMR unvaccinated group. As for comorbidities, children were less likely to receive the MMR vaccine if they were born with a congenital anomaly (2.12; 1.90 to 2.36), cancer (4.89; 3.97 to 6.01), never admitted to an ICU (1.88; 1.78 to 1.98) or never visited an ER (3.57; 3.53 to 3.72).

## 4. Discussion

In this nationwide study, we found substantial differences in both the parental and clinical characteristics between the MMR vaccinated and unvaccinated groups among children born between 2008 and 2016 in South Korea. Children who resided in a metropolitan area, beneficiaries of a self-employed health insurance and those with low household income level were less likely to receive the MMR vaccine. Healthcare underutilization was also evident in these children, and clinical factors such as congenital anomaly and cancer diagnosis had a major negative impact on the MMR vaccine uptake. Notwithstanding the fact that the overall vaccination coverage of the MMR vaccine in South Korea is sufficient to obtain herd immunity, the number of MMR unvaccinated children showed an increasing trend throughout the study period.

Factors that may have an impact on vaccination such as concerns about adverse events following immunization, insufficient knowledge about the vaccine, appraisal of illness and general attitudes toward the vaccine have been reviewed previously [14]. Amongst these factors, the health status of children was identified as one of the outstanding factors associated with vaccine hesitancy; Taylor and colleagues found that children often acutely ill to received vaccines were more likely to be under-vaccinated (relative risk 2.37, 95% CI 2.07 to 2.68) [15], and Deborah and colleagues also identified children’s acute illness as the major factor delaying vaccination [16]. Indeed, some of the comorbidities, such as cancer and congenital anomaly, were associated with MMR unvaccination in our study. These comorbidities are closely related to childhood health and are likely to be a barrier to timely vaccination. Specifically, childhood cancer is one of the leading causes of death and requires specialized medical interventions tailored to each child’s prognosis [17]. In this regard, routine vaccination can be easily delayed or even omitted in this subset while on intensive chemotherapy followed by variable degree of immune suppression [18]. Children born with congenital anomaly are also likely to be receiving specialized care, and parents or caregivers may be too overwhelmed by the clinical complexity to keep up with the routine childhood vaccination. Conversely, several adverse pregnancy outcomes including birth injury, infections in the perinatal period, a low birth weight and being small for the gestational age were inversely associated with MMR unvaccination. These conditions are acute complications at birth and less likely to impact the child’s health status at the time of vaccination [19]. Although several studies found that the sex of the child was significantly associated with being unvaccinated [7,20], others did not show significant results [8,21]. In our study, girls were more likely to be unvaccinated, but the finding should be interpreted with caution considering the point estimate, which is very close to 1, and the confidence interval. A further study would be needed to identify association between sex and vaccination.

Of the parental factors, inconsistent findings have been observed for the association between the household income and vaccine uptake. One study in Canada that analyzed administrative health data found that a higher parental socioeconomic status was associated with incomplete vaccination, assessed up to 24 months of birth [22], and another study in Brazil reported that the coverage of the recommended vaccines until 18 months of birth was 77.2% in the highest socioeconomic stratum and 86.2% in the lowest stratum [23]. Conversely, an interview-based study in China showed a declining trend of immunization coverage with a lower income level [24], which was also evident in a nationwide cohort study conducted in the United Kingdom that reported that vaccine uptake increased with the household income [7]. Our study finding on the association between a low household income level and unvaccination is in line with the trends observed in the latter studies. With regard to healthcare utilization and vaccine uptake, a recent study from the Vaccine Safety Datalink (VSD) project identified intentional vaccine refusers from an immunization registry and reported that this subset of children aged less than two years had fewer hospital days and were less likely to be seen in outpatient settings or ERs than nonrefusers [25]. Similarly, the MMR unvaccinated group in our study demonstrated less frequent outpatient and ER visits compared with the vaccinated group. Of note, we also included Korean traditional medicine clinic visits as a potential factor for unvaccination based on the finding from a past study that revealed trust in Korean traditional medicine or alternative medicine as one of the reasons for vaccine hesitancy [26]. While our study found null association between Korean traditional medicine clinics and unvaccination, this does not necessarily preclude its association with vaccine hesitancy or delay as our finding is only relevant for the uptake of the MMR vaccine. 

Overall, by using a nationwide database with a high generalizability, our study ascertained findings from past studies that were either limited due to a small sample size or where the methodological design was susceptible to recall bias. However, several limitations should be noted in interpreting this study. First, there is an inherent limitation in the use of a nationwide administrative health database as some characteristics such as the use of antenatal care, birth order, educational status, marital status, religion and reasons for unvaccination are not recorded. Second, potential misclassifications may be presented. Immunization records that are covered under the National Immunization Program are included in the KIRIS, and thus if a child were vaccinated outside the country or his/her parent intentionally paid out of pocket, such a record would not be available. Moreover, there is no legal duty for healthcare practitioners to enter immunization records into the system, and the Korea Disease Control and Prevention Agency does not enforce against such negligence [6]. With regard to misclassification of the covariates, comorbidities are unlikely to be captured if a person does not utilize healthcare resources. Last, the month and date of birth was omitted, and only the age in years at vaccination was available in our data source due to the personal information protection act. this limitation inherently led us to assign a fixed month and date (i.e., July 1st) and subsequent year of birth (i.e., index year of 2010 for a child born in 2009) for the index date of the MMR unvaccinated group. However, the factors associated with MMR vaccine uptake assessed in our study were mostly time-independent and thus unlikely to have an impact on our study findings.

## 5. Conclusions

In this nationwide register-based study, considerable differences were observed between the MMR vaccinated and unvaccinated groups. Children’s clinical factors, as well as parental socioeconomic factors, were associated with unvaccination of MMR. These findings can contribute to improving MMR vaccination coverage and to recommending a timely vaccination, underscoring the need to optimize targeted interventions tailored to the subset of children in South Korea.

## Figures and Tables

**Table 1:** Characteristics by MMR vaccination status among children born between January 2008 and December 2016.

Characteristic	MMR Vaccinated (*n* = 3,897,579)		MMR Unvaccinated (*n* = 75,674)	
	N	(%)	N	(%)
**Sex**				
Boys	2,001,404	(51.3)	37,633	(49.7)
Girls	1,896,175	(48.7)	38,041	(50.3)
**Parental socioeconomic status**				
Residence type ^†^				
Metropolitan	1,738,321	(44.6)	37,923	(50.1)
Provincial	2,159,258	(55.4)	37,751	(49.9)
Type of health insurance				
Employer-sponsored	2,652,840	(68.1)	21,097	(27.9)
Self-employed	783,171	(20.1)	43,728	(57.8)
Medical aid beneficiary	32,872	(0.8)	377	(0.5)
Unknown	428,696	(11.0)	10,472	(13.8)
Household income level				
Low	286,376	(7.3)	15,979	(21.1)
Lower-Middle	561,145	(14.4)	11,903	(15.7)
Upper-Middle	1,249,361	(32.1)	14,145	(18.7)
High	1,295,646	(33.2)	22,474	(29.7)
Unknown	505,051	(13.0)	11,173	(14.8)
**Comorbidity**				
Birth injury	45,031	(1.16)	292	(0.39)
Congenital anomaly	14,395	(0.37)	370	(0.49)
Infections in perinatal period	535,422	(13.7)	3311	(4.38)
Low birth weight	161,489	(4.14)	1139	(1.51)
Small for gestational age	13,111	(0.34)	104	(0.14)
Asplenia	1903	(0.05)	26	(0.03)
History of anaphylaxis	1317	(0.03)	16	(0.02)
Prior MMR infection	6927	(0.18)	94	(0.12)
Cancer	1863	(0.05)	107	(0.14)
**Healthcare utilization**				
Outpatient visit (mean ± SD)	25.8 ± 17.06		5.73 ± 12.1	
Hospitalization days (mean ± SD)	2.32 ± 6.90		1.69 ± 14.5	
ICU visit (mean ± SD)	0.06 ± 0.33		0.04 ± 0.42	
Yes	177,865	(4.56)	1468	(1.94)
No	3,719,714	(95.4)	74,206	(98.06)
ER visit (mean ± SD)	0.46 ± 0.90		0.15 ± 0.71	
Yes	1,049,688	(26.9)	6532	(8.63)
No	2,847,891	(73.1)	69,142	(91.4)
Korean traditional medicine clinic visit (mean ± SD)	0.33 ± 2.37		0.34 ± 3.88	
Yes	298,356	(7.6)	5028	(6.6)
No	3,599,223	(92.4)	70,646	(93.4)

Abbreviations: MMR, Measles-Mumps-Rubella; ICU, intensive care unit; ER, emergency room; SD, standard deviation. ^†^ Metropolitan, cities with a population of over 1 million.

**Table 2:** Multivariate logistic regression analysis of the factors associated with MMR unvaccination among children born between January 2008 and December 2016.

Characteristic	aOR *	95% CI
**Sex**		
Boys	Ref	
Girls *	1.02	(1.00 to 1.03)
**Parental socioeconomic status**		
Residence type ^†^		
Metropolitan *	1.44	(1.41 to 1.46)
Provincial	Ref	
Type of health insurance		
Employer-sponsored	Ref	
Self-employed *	7.34	(7.21 to 7.46)
Medical aid beneficiary *	0.43	(0.39 to 0.48)
Household income level		
Low *	3.66	(3.58 to 3.74)
Lower-Middle	0.98	(0.96 to 1.01)
Upper-Middle *	0.62	(0.61 to 0.64)
High	Ref	
**Comorbidity**		
Birth injury *	0.38	(0.34 to 0.43)
Congenital anomaly *	2.12	(1.90 to 2.36)
Infections in perinatal period *	0.32	(0.31 to 0.33)
Low birth weight *	0.42	(0.39 to 0.44)
Small for gestational age *	0.63	(0.51 to 0.76)
Asplenia	0.88	(0.59 to 1.30)
History of anaphylaxis	1.43	(0.87 to 2.36)
Prior MMR infection	1.07	(0.87 to 1.32)
Cancer *	4.89	(3.97 to 6.01)
**Healthcare utilization**		
ICU visit		
Yes	Ref	
No *	1.88	(1.78 to 1.98)
ER visit		
Yes	Ref	
No *	3.57	(3.53 to 3.72)
Korean traditional medicine clinic visit		
Yes	Ref	
No	0.99	(0.96 to 1.02)

Abbreviations: MMR, Measles-Mumps-Rubella; aOR, adjusted odds ratio. * Characteristics indicating a statistically significant odds ratio. ^†^ Metropolitan, cities with a population of over 1 million.

## Data Availability

We used the customized data extracted and provided by the National Health Insurance Sharing System (Further details available at: https://nhiss.nhis.or.kr/bd/ab/bdaba032eng.do (accessed on 21 December 2020)).

## Supplementary Materials

The following are available online at https://www.mdpi.com/article/10.3390/vaccines9060653/s1, Table S1: Diagnosis codes for defining comorbidities, Figure S1 (**a**): Coverage of other routinely recommended vaccines by the first year of birth among the MMR unvaccinated group born between 2008 and December 2016, Figure S1 (**b**): Coverage of other routinely recommended vaccines by the first year of birth among the MMR vaccinated group born between 2008 and December 2016.
