# Supplementary material for: Differential Demographic and Clinical Characteristics between MMR Vaccinated and Unvaccinated Children in South Korea: A Nationwide Study
Source: Vaccines (Basel). 2021 Jun 15;9(6):653. doi: 10.3390/vaccines9060653 (PMC8232726; doi:10.3390/vaccines9060653)
Supplement: Supplementary file 1 [file vaccines-09-00653-s001.zip › vaccines-1209717-supplementary.pdf]

Table S1. Diagnosis codes for defining comorbidities

Conditions	ICD-10 code(s)
Small for gestational age	P051
	C00-C97, D00-D48
Low birthweight	P07 or catastrophic illness domestic code (F002)
Birth injury	P10-P15
Infections in perinatal period	P45-P39
Congenital anomaly	Q00-Q99 (Q89 excluded) or catastrophic illness domestic codes (V021, V143, V144, V145, V146, V147, V148, V149, V150, V151, V154, V155, V156, V157, V158, V159, V160, V179, V180, V181, V182, V183, V184, V185, V186, V204, V205, V214, V215, V216, V217, V218, V225, V226, V227, V228, V229, V230, V239, V240, V241, V242, V243, V244, V245, V264, V265, V266, V267, V269, V270, V271, V272)
Asplenia	Q89
Anaphylaxis	T78.2, T88.6
Measles	B05
Mumps	B25
Rubella	B06

ICD, International Classification of Disease

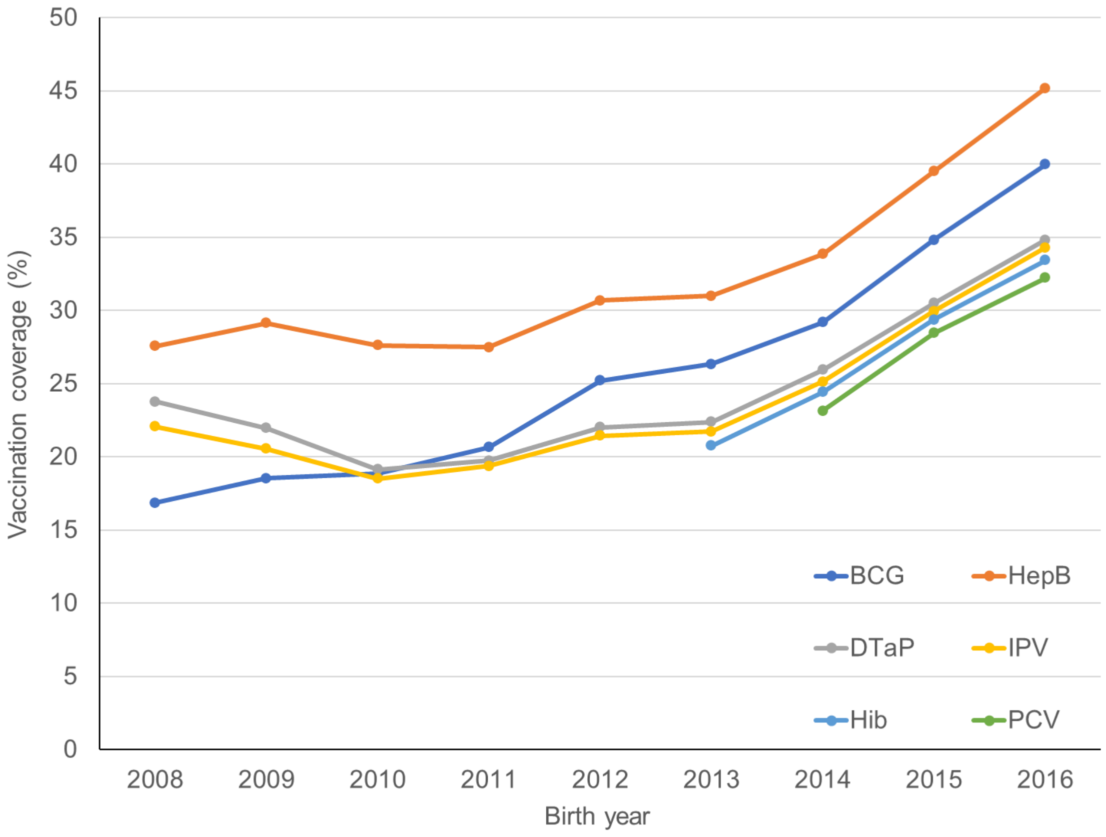

Figure S1(a). Coverage of other routinely recommended vaccines by the first year of birth among MMR unvaccinated group born between 2008 and Dec 2016.

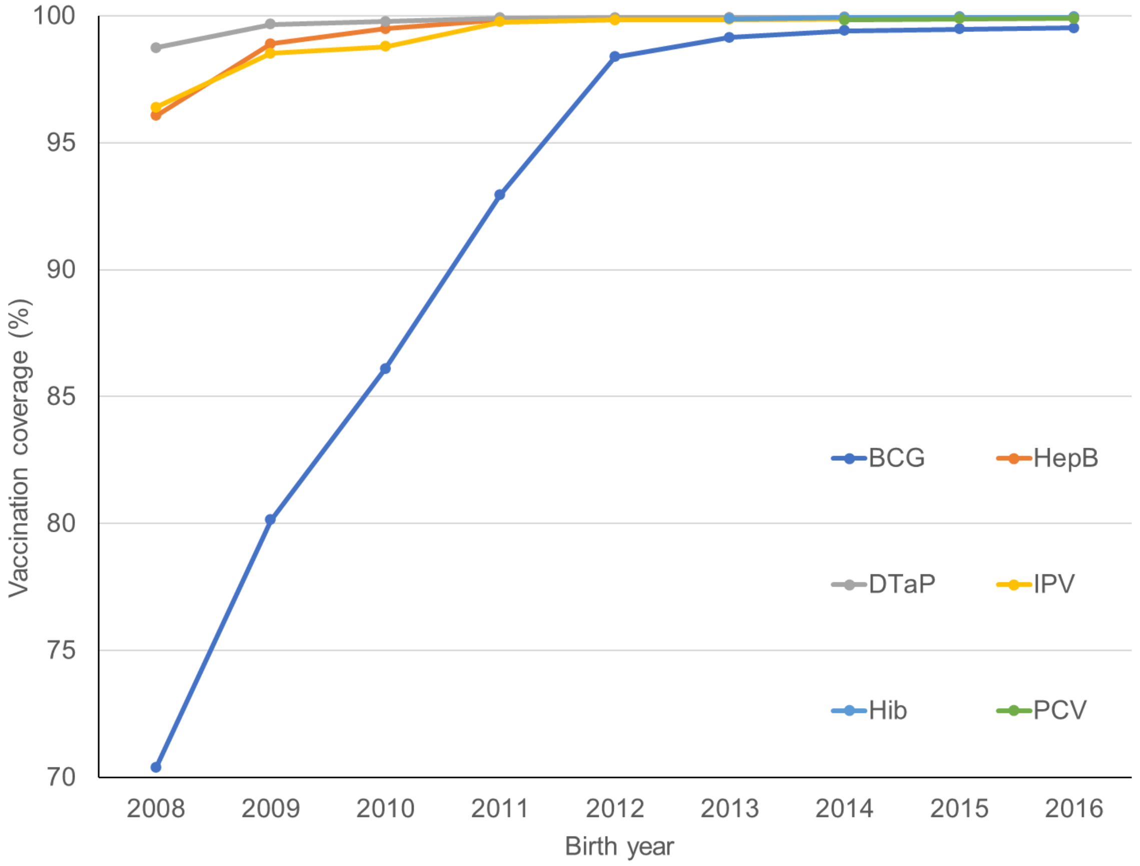

Figure S1(b). Coverage of other routinely recommended vaccines by the first year of birth among MMR vaccinated group born between 2008 and Dec 2016.
